# Exploring research trends and hotspots on PCSK9 inhibitor studies: a bibliometric and visual analysis spanning 2007 to 2023

**DOI:** 10.3389/fcvm.2024.1474472

**Published:** 2024-11-22

**Authors:** Ping Lai, Shuquan Xu, Ziyou Liu, Jiayuan Ling, Kejun Tian, Jianwei Yan, Dong Chen, Yiming Zhong, Jinhua Xue

**Affiliations:** ^1^ Department of Cardiology, First Affiliated Hospital of Gannan Medical University, Gannan Medical University, Ganzhou, Jiangxi, China; ^2^ Key Laboratory of Prevention and Treatment of Cardiovascular and Cerebrovascular Diseases, Ministry of Education, Gannan Medical University, Ganzhou, China; ^3^ The First School of Clinical Medicine, Gannan Medical University, Ganzhou, China; ^4^ Department of Heart Center, First Affiliated Hospital of Gannan Medical University, Ganzhou, China; ^5^ Department of Physiology, School of Basic Medicine, Gannan Medical University, Ganzhou, China

**Keywords:** proprotein convertase subtilisin/kexin type 9 (PCSK9), lipid management, cardiovascular disease, emerging research hotspots, bibliometric analysis

## Abstract

**Background:**

Following the identification of proprotein convertase subtilisin/kexin type 9 (PCSK9) inhibitors, research in this area has experienced significant growth. However, a thorough bibliometric analysis of this burgeoning field remains conspicuously absent. The current study aims to delineate research hotspots and anticipate future trends on PCSK9 inhibitors employing bibliometric analysis.

**Methods:**

A systematic search was conducted in the Web of Science Core Collection (WoSCC) to identify scholarly articles and reviews pertaining to PCSK9 inhibitors, yielding 1,812 documents. Data extraction was followed by analysis and visualization using Excel, VOSviewer, and CiteSpace software.

**Results:**

A total of 1,812 publications were included in the final analysis. Ray, KK from the UK was the most prolific author, followed by Pordy, R from the USA. The USA led in publication output [number of publications (Np):776] and number of citations without self-citations (Nc) at 34,289, as well as an H-index of 93. “Cardiovascular System Cardiology” emerged as the predominant subject area. Amgen and the Journal of Clinical Lipidology were identified as the most active institution and journal, respectively. Keywords such as “lipoprotein(a),” “bempedoic acid,” “percutaneous coronary intervention,” “inclisiran,” “peripheral artery disease,” “mortality,” and “endothelial dysfunction” are gaining prominence in the field.

**Conclusion:**

The research on PCSK9 inhibitors is experiencing a sustained growth trajectory. The USA exerts considerable influence in this area, with the Journal of Clinical Lipidology expected to feature more groundbreaking studies. Research on “lipoprotein(a)”, “bempedoic acid”, “percutaneous coronary intervention”, “peripheral artery disease”, and “endothelial dysfunction” are poised to become focal points of future investigation.

## Introduction

Proprotein convertase subtilisin/kexin type 9 (PCSK9) is a protein secreted by the liver that plays a critical role in regulating low-density lipoprotein cholesterol (LDL-C) levels by modulating the degradation of the low-density lipoprotein receptor (LDLR), and PCSK9 inhibitors have emerged as a groundbreaking class of therapeutic agents in the field of lipid management (1). Historically, the discovery of PCSK9 as a key regulator of LDL-C clearance opened new avenues for addressing dyslipidemia and cardiovascular disease (CVD) (2, 3). Early 2000s genetic studies identified loss-of-function mutations in PCSK9 associated with reduced LDL-C levels and a decreased risk of cardiovascular events, underscoring its therapeutic potential (3). Subsequent research culminated in the development of PCSK9-targeting monoclonal antibodies, marking a milestone in drug design (4).

PCSK9 inhibitors act by blocking the interaction between PCSK9 and LDLR, leading to increased recycling of LDLRs and enhanced clearance of LDL-C (5). This mechanism significantly amplifies the liver's LDL-C removal capacity, surpassing the effects of statins and other lipid-lowering therapies (6). This unique mechanism makes PCSK9 inhibitors a valuable addition to the treatment arsenal for managing dyslipidemia, especially in patients who have not reached target LDL-C levels with standard therapies (7). With robust evidence from clinical trials, PCSK9 inhibitors have demonstrated their efficacy in reducing LDL-C levels and cardiovascular events (8). Additionally, Ongoing research is exploring the use of PCSK9 inhibitors in various patient populations, including those with diabetes (9, 10), chronic kidney disease (11), and refractory dyslipidemia (12).

As PCSK9 inhibitors continue to gain acceptance in clinical practice, real-world data analyses have further supported their long-term safety and efficacy profiles (13). Observational studies and post-marketing surveillance efforts provide valuable insights into the practical clinical utility and benefits of PCSK9 inhibitors in routine clinical settings (14). Moreover, ongoing trials are investigating novel PCSK9 inhibitor formulations, dosing strategies, and combination therapies to optimize their lipid-lowering effects and further reduce cardiovascular risk (10, 15). As research continues to unravel the full potential of PCSK9 inhibitors, their role in reducing cardiovascular risk is likely to be further refined, ultimately enhancing long-term outcomes and the overall well-being of patients with dyslipidemia and CVD.

Given the rapid research expansion in PCSK9, a bibliometric analysis of the field remains conspicuously absent. This study aims to provide researchers with a comprehensive overview of the research background and status of the PCSK9 inhibitor field. In this current study, we employ bibliometric analysis to unveil the landscape of studies, focal points of research, and potential avenues of investigation within the area of PCSK9 inhibitor which lays the groundwork for future research initiatives.

## Materials and methods

### Search strategy

**Figure 1:**
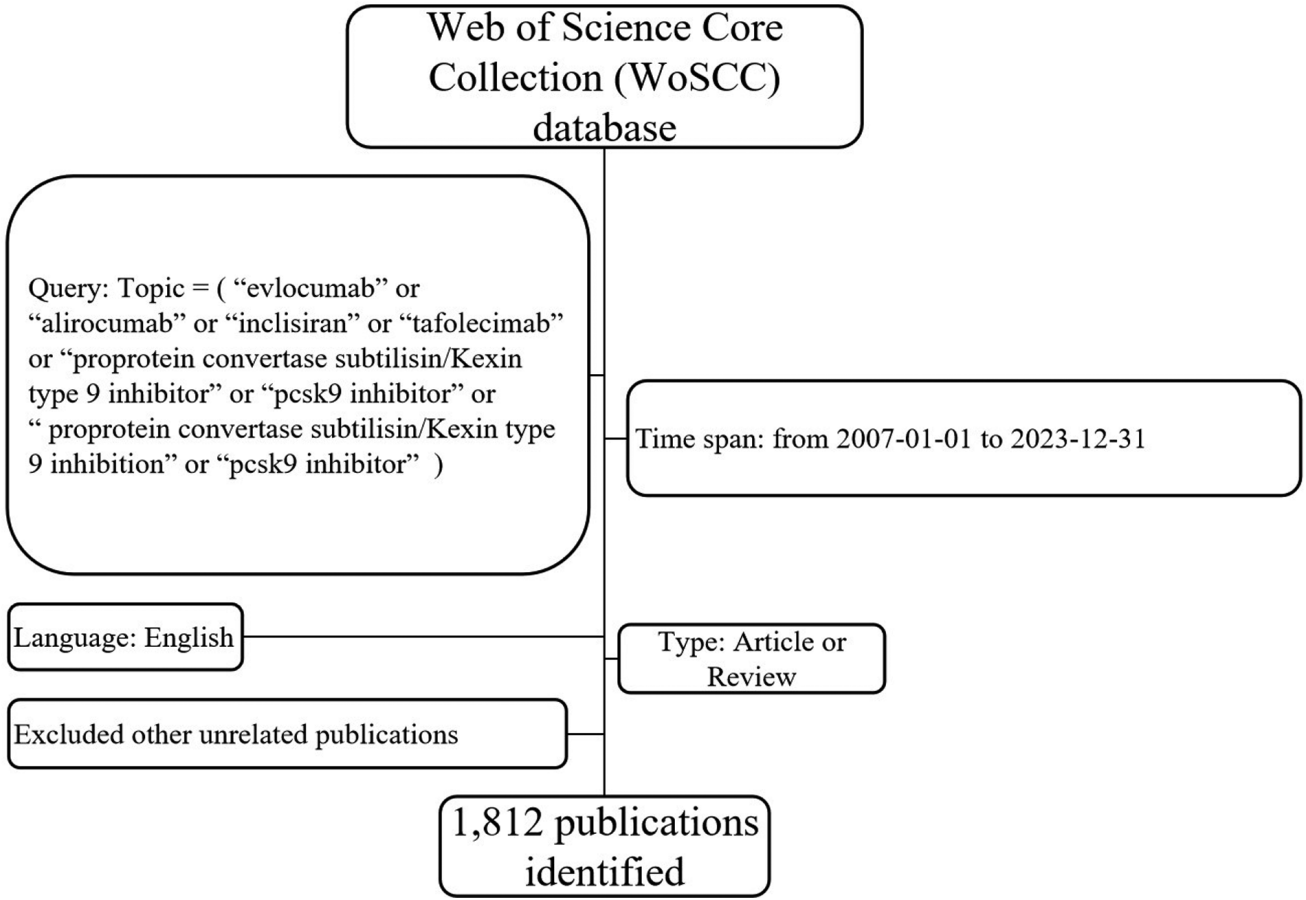
Flow chart of current bibliometric analysis.

On January 31, 2024, the research criteria were set as follows: the search terms “evolocumab” or “alirocumab” or “inclisiran” or “tafolecimab” or “proprotein convertase subtilisin/kexin type 9 inhibitor” or “pcsk9 inhibitor” or “proprotein convertase subtilisin/kexin type 9 inhibition” were included in the topic. The search was conducted using the Web of Science core collection (WoSCC). The published time was set from 1 January 2007 to 31 December 2023. A total of 2,799 papers were searched, including letters, meeting abstracts, editorials, preprints, and other types of publications. After filtering for articles and reviews in English, 1,812 publications were selected for analysis. The full records, containing information such as authors, titles, publication dates, journal titles, institutions, keywords, citations, funding, and references, were exported as plain text files. The flow chart depicting the search process can be found in Figure 1. Additionally, the 2022 impact factor and the Hirsch index (H-index) were extracted directly from the WoSCC.

### Data analysis

Excel (Microsoft 97-2003) was utilized to analyze various bibliometric indicators such as the number of publications, citations, countries or regions, top authors, research institutions, funding sources, research areas, and journals. The average citation number (ACN) was calculated by dividing the total number of citations without self citation (Nc) to the total number of publications (Np). The H-index, provided by WoSCC, was used to assess the impact of authors.

VOS viewer, a JAVA-based software (version 1.6.18, CWTS, Leiden University) (link: https://www.vosviewer.com/download), was utilized to create network visualizations for authors, countries and cited publication (16). CiteSpace (version 6.2.R4) (link: https://sourceforge.net/projects/citespace/files/) developed by Chaomei Chen was employed for detecting clusters of keywords from publications with high citation bursts, and creating the visual map of the keyword network from the timeline view (17).

Clusters of keywords from publications with high citation bursts were identified, and a timeline-based keywords network visualization was generated using CiteSpace. The CiteSpace parameters were configured as follows: time span (2007–2023), years per slice (1 year), term source (title, abstract, author keywords, and keyword plus), node type (keyword), link strength (cosine), and scope (within slices). The selection criteria applied the g-index (k = 25), with pruning techniques including minimum spanning tree, pruning sliced networks, and pruning the merged network. The log-likelihood ratio (LLR) method was used for clustering, with all clusters labeled by relevant keywords.

## Results

### The trend of publications and citations over time

**Figure 2:**
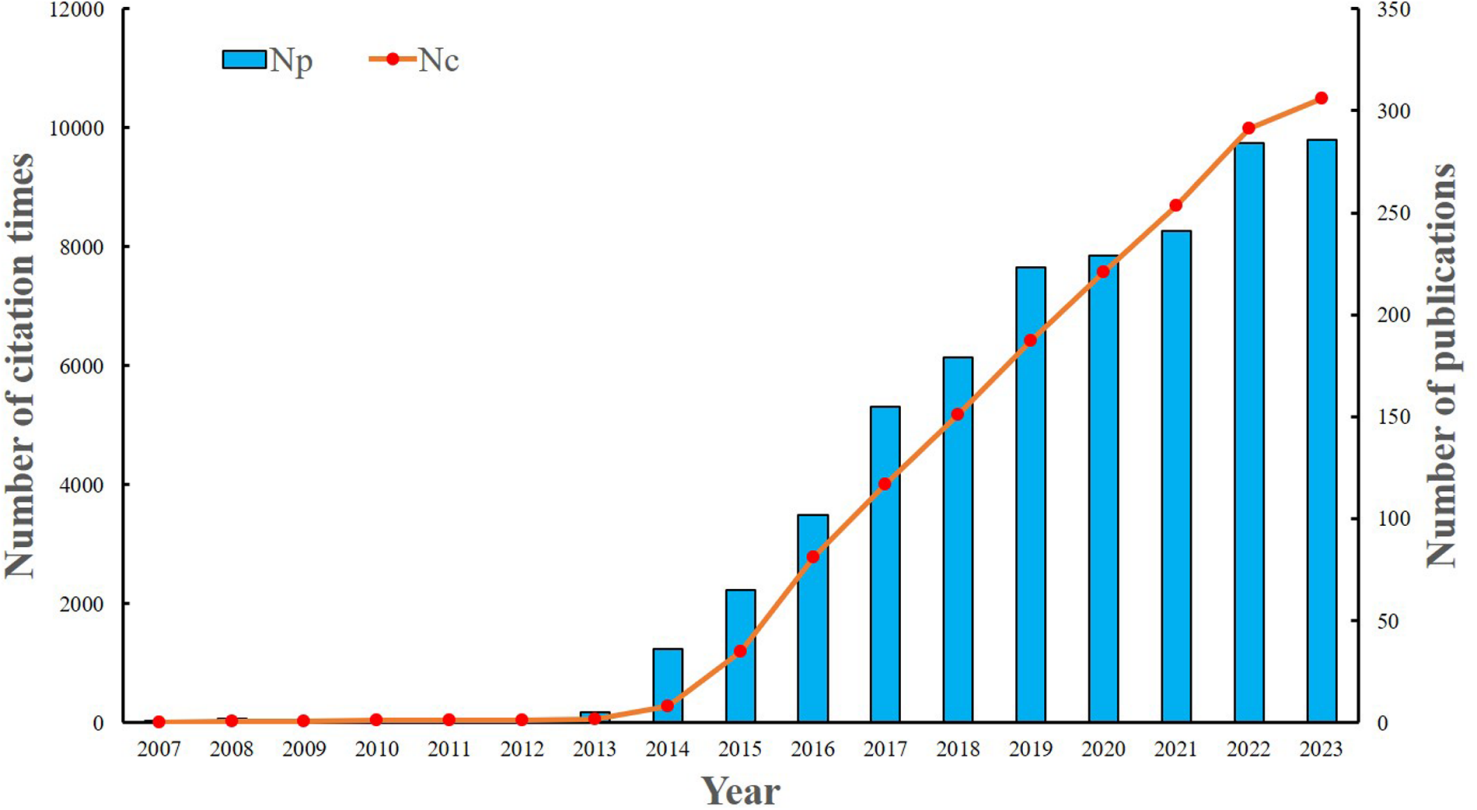
The number of annual publications and citations from 2007 to 2023.

A total of 1,812 publications were identified using the search strategy in the Web of Science Core Collection (WoSCC). Among these, 1,245 (68.7%) were articles, while 567 (31.2%) were reviews. Initially, there was limited research focus in this area. However, a consistent upward trend has been observed since 2013. Between 2014 and 2023, a total of 1,800 articles were published, representing 99.3% of the overall articles. Excluding self-citations, the 1,812 articles amassed a total of 56,857 citations, yielding an average of 33.3 citations per article (Figure 2).

### Analysis of primary authors

**Figure 3:**
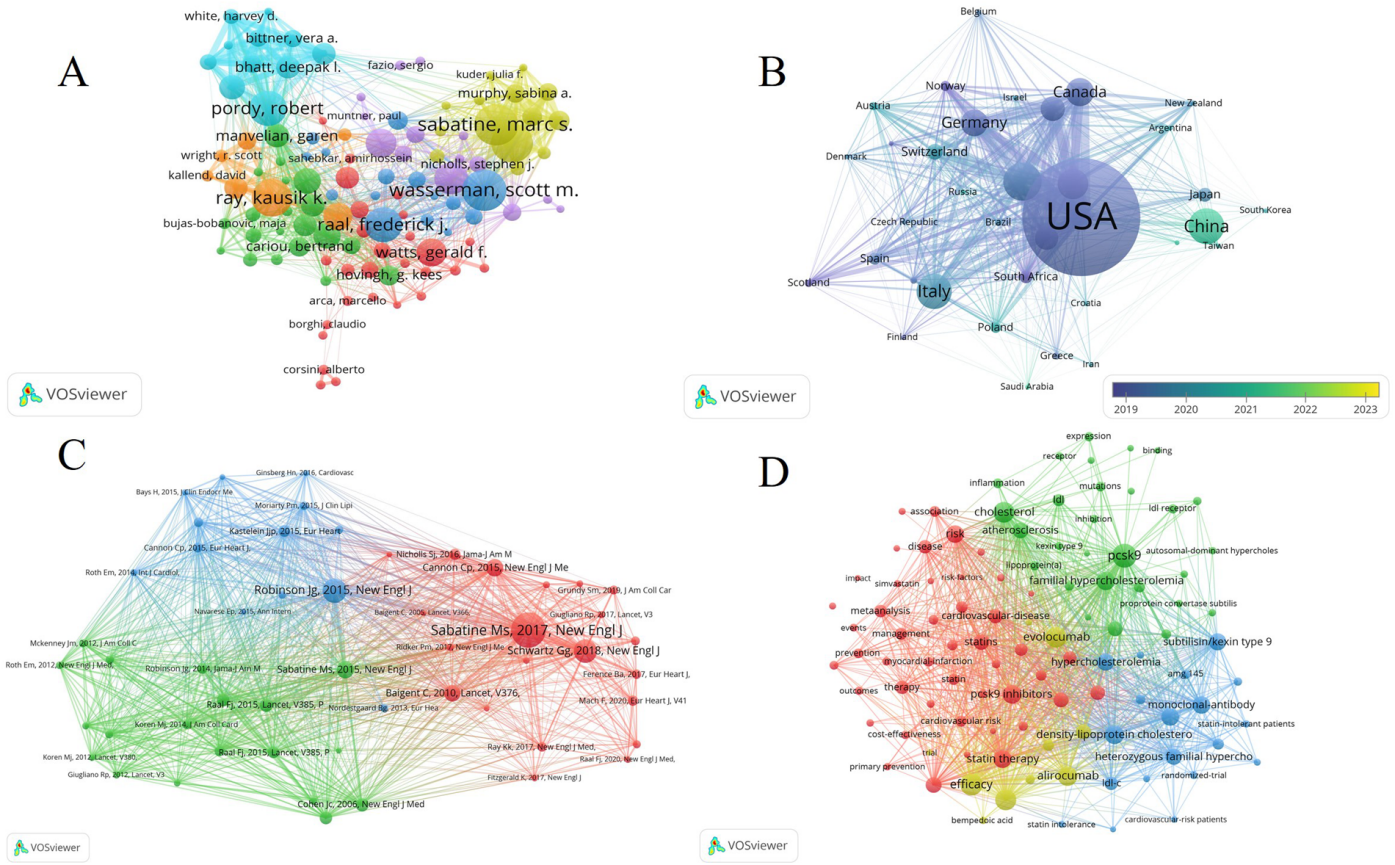
**(A)** Visual network map of 109 authors with more than 10 publications. **(B)** Visual network of a country or region with more than 15 publications. **(C)** Analysis of the co-citation network of cited publications. **(D)** Bibliographic analysis of keywords with more than 50 occurrences.

The top ten most productive authors collectively contributed 484 articles, comprising 26.7% of all publications. Notably, Ray, KK (Np: 62, Nc: 6,137, H-index: 32) from the UK emerged as the most prolific author in this area. Following closely, Pordy, R, from the USA, secured the second position with 55 publications. Wasserman, SM (Np: 53, Nc: 12,033, H-index: 37), though ranking fourth in terms of article output, claimed the top spot in H-index. These top ten authors hailed from diverse countries, including the USA (7), UK (1), South Africa (1), and Netherlands (1) (Supplementary Table S1). Moreover, a total of 109 authors in this field had over ten publications. Figure 3A visually depicts the significant contributions of core authors, with Wasserman, SM, Ray, KK, and Pordy, R, prominently featured.

### Analysis of influential countries/subjects/institutions/journals

The top ten prolific countries based on Np, with the USA leading with the highest number of articles (Np: 776, Nc: 34,289, H-index: 93), followed by England (Np: 260, Nc: 18,659, H-index: 60), and China (Np: 244, Nc: 4,620, H-index: 28). Notably, the USA also holds the highest citations and H-index among these countries, reflecting its prominent position in the field of PCSK9 inhibitors (Supplementary Table S2).

Furthermore, the top ten most productive subjects in this field in terms of the number of publications is leaded by “Cardiovascular System Cardiology” (Np: 812, Nc: 23,81, H-index: 81), and followed by “Pharmacology Pharmacy” (Np: 483, Nc: 6,218, H-index: 388), and “General Internal Medicine” (Np: 191, Nc: 15,950, H-index: 33). These findings provide valuable insights into the primary areas of research focus within the domain of PCSK9 inhibitors (Supplementary Table S2).

Among the 35 countries or regions that contributed more than 15 publications, the USA exhibited the closest collaborations with other countries, underscoring its leadership in this field. Notably, China emerges as the most recent major producer of publications in this domain (Figure 3B).

In terms of the top 10 institutions with the highest number of publications related to this area, Amgen (Np: 164, Nc: 15,426, H-index: 54) had the highest Np, followed by Harvard University (Np: 161, Nc: 16,089, H-index: 53). Five of the top ten institutions in this field are in USA, and three are in the France (Supplementary Table S3).

Regarding journals, the Journal of Clinical Lipidology (Np: 87, H-index: 25) emerged as the leading publisher in this field, followed by Atherosclerosis (Np: 48, H-index: 18), and the Journal of The American College of Cardiology (Np: 44, H-index: 28). Collectively, papers published in the top ten academic journals constituted 22.07% of all papers. Notably, the Journal of The American College of Cardiology, despite ranking third in the number of publications, had the highest Nc among the top journals (Supplementary Table S3).

### Highly influence publication

The top ten most cited publications are shown in Supplementary Table S4, and all top ten cited publications were published after 2015. The top cited paper was an article published in the journal of the *New England Journal of Medicine* titled “Evolocumab and Clinical Outcomes in Patients with Cardiovascular Disease” in 2017 by Sabatine, MS et,al. The study, a randomized, double-blind, placebo-controlled trial, found that Evolocumab, which inhibits proprotein convertase subtilisin/kexin type 9 (PCSK9) and lowers low-density lipoproteins (LDL), was effective in patients with atherosclerotic cardiovascular disease (ACVD), reducing median LDL cholesterol levels to a median of 30 mg per deciliter (0.78 mmol per liter) and reducing the risk of cardiovascular events.

The paper “2018 AHA/ACC/AACVPR/AAPA/ABC/ACPM/ADA/AGS/APhA/ASPC/NLA/PCNA Guideline on the Management of Blood Cholesterol: Executive Summary” was the second highest cited paper, which was published in Journals of the American College of Cardiology by Grundy, SM et al. in 2019. This paper highlights the correlation between cholesterol levels and the risk of ACVD, and offers guidance for the formulation of combined lipid-lowering therapy. It suggests that the appropriate addition of PCSK9 inhibitors may enhance lipid-lowering efficacy. However, it cautions that the economic value of PCSK9 inhibitor therapy must be carefully evaluated before widespread adoption.

The third highest cited paper was “Alirocumab and Cardiovascular Outcomes after Acute Coronary Syndrome” from *New England Journal of Medicine* by Schwartz, GG et,al. in 2018. This investigation, conducted within a multicenter, randomized, double-blind, placebo-controlled trial framework, found that among patients who had experienced acute coronary syndromes and were treated with high-dose statin therapy, treatment with alirocumab, a PCSK9 inhibitor, was associated with a reduced risk of recurrent ischemic cardiovascular events compared to placebo. These findings imply that PCSK9 inhibitors may offer superior clinical utility.

To show the relationship between those high cited papaers, a co-citation map of cited publications with over 120 citations performed by vosviewer (Figure 3C).

### Analysis of keywords

Keywords were extracted from all 1,812 publications for co-occurrence analysis by CiteSpace. Among the top twenty frequency keywords, “efficacy,” “safety,” “density lipoprotein cholesterol,” “monoclonal antibody,” and “evolocumab” ranked first to fifth with a frequency of 495, 440, 353, 324, and 316, respectively (Supplementary Table S5).

In Figure 3D, we present an analysis of keywords with more than 50 times. All keywords were categorized into 10 clusters, each named after the first keyword in that group. Among them, the top three clusters were “PCSK9,” “Acute Coronary Syndrome,” and “Familial Hypercholesterolaemia” (Supplementary Table S6). It's worth noting that the size of the clusters may increase with the expansion of cluster labels.

**Figure 4:**
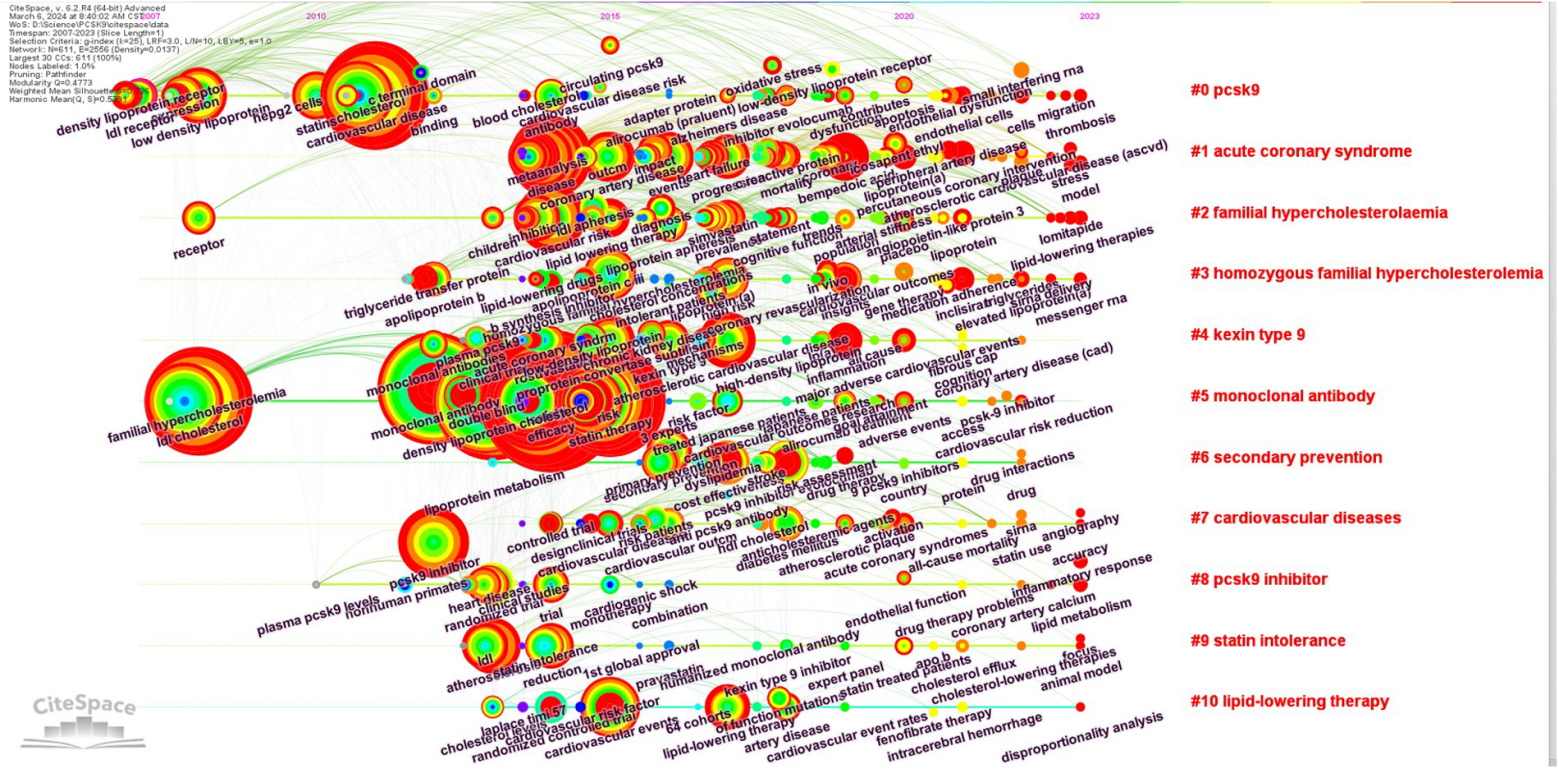
Visual map of the evolution of keywords from the main clusters.

To understand the evolution of keywords, a visual timeline of the keywords in the clusters was constructed spanning from 2007 to 2023. Notable terms such as “endothelial dysfunction,” “peripheral artery disease,” “percutaneous coronary intervention,” and “inclisiran” were extensively researched during this period (Figure 4).

For further insights into the research frontiers, the top 50 keywords with the highest burst intensity and burst year were identified using CiteSpace (Supplementary Table S7). Emerging keywords include “lipoprotein(a)” (strength 3.49, 2020–2023), “bempedoic acid” (strength 10.59, 2021–2023), “percutaneous coronary intervention” (strength 4.85, 2021–2023), “inclisiran” (strength 4.55, 2021–2023), “peripheral artery disease” (strength 4.37, 2021–2023), “mortality” (strength 3.75, 2021–2023), and “endothelial dysfunction” (strength 3.50, 2021–2023). These keywords signify the increasing significance and recent focus on these specific aspects within the field.

## Discussion

In this research, we conducted a thorough bibliometric analysis to assess the PCSK9 inhibitor literature, identifying key trends, prolific authors like Ray, KK from the UK, and the USA's leading role in publication and citation metrics. Our analysis also highlighted influential entities and journals, providing a framework for future research directions within this scientific domain.

PCSK9 inhibitors represent a breakthrough in lipid-lowering therapy, with considerable implications for CVD management (2). By targeting PCSK9, these agents enhance the hepatic uptake of low-density lipoprotein cholesterol (LDL-C), thereby significantly reducing circulating LDL-C levels (18). This mechanism of action positions PCSK9 inhibitors as a promising adjunct to traditional statin therapy. The role of PCSK9 inhibitors extends beyond LDL-C reduction, as they also modulate levels of lipoprotein(a) [Lp(a)] (19). Recent studies have begun to reveal the impact of PCSK9 inhibitors on Lp(a) levels, offering a new therapeutic approach for patients with elevated Lp(a) levels (20). Elevated Lp(a) is linked to higher risks of coronary artery disease, myocardial infarction, and stroke, and managing it presents a clinical challenge, especially in patients with limited responses to statins (21). Currently, clinical trials have observed a modest reduction in Lp(a) levels due to PCSK9 inhibitors (22). Although the reduction in Lp(a) is modest compared to LDL-C, it could translate into meaningful cardiovascular risk reduction, especially in patients with high Lp(a) (23). The mechanisms underlying PCSK9 inhibitors' impact on Lp(a) are not yet fully elucidated, highlighting a significant gap in our understanding (24). The mechanisms underlying PCSK9 inhibitors' impact on Lp(a) are not yet fully elucidated, highlighting a significant gap in our understanding.

Bempedoic Acid (BA) has emerged as a novel oral lipid-lowering agent that targets cholesterol biosynthesis in the liver, thereby reducing low-density lipoprotein cholesterol (LDL-C) levels through the inhibition of ATP citrate lyase (25). This mechanism distinguishes BA from statins, as it is inactive in muscle tissue, which may translate to a lower risk of myopathy and other muscle-related side effects commonly associated with statin use (26, 27). This characteristic renders BA a viable alternative for patients with hyperlipidemia or ASCVD who are statin-intolerant or require more aggressive LDL-C reduction. The potential of combining BA with PCSK9 inhibitors has been a recent focus, as both drugs lower LDL-C through distinct mechanisms, suggesting a possible synergistic effect (28). Especially, a novel small interfering RNA (siRNA) therapy that targets the PCSK9 gene like inclisiran, reducing PCSK9 gene expression at the mRNA level. Administered via subcutaneous injection twice a year, inclisiran provides sustained LDL-C reduction by decreasing circulating PCSK9 levels, resulting in enhanced LDL receptor recycling. Inclisiran is especially beneficial for patients with familial hypercholesterolemia and those at high cardiovascular risk who require consistent, long-term lipid management.

Moreover, the combined use of these drugs may provide a synergistic effect in reducing the risk of cardiovascular events (29). Early research indicates that this combination may achieve additional LDL-C reduction without exacerbating side effects, providing a promising therapeutic strategy for patients who do not meet LDL-C targets with monotherapy (30, 31). These findings provide new strategies for cardiovascular risk management, especially for patients who respond poorly to conventional treatment methods. While the combination of PCSK9 inhibitors and BA shows promise, it is imperative to continue assessing the safety and tolerability profile in long-term studies (32). Although current evidence suggests that the combination is well-tolerated, a comprehensive evaluation of its safety over extended use is necessary (33). PCSK9 inhibitors and BA demonstrate individual benefits in lowering LDL-C levels and managing CVD risk. While these drugs act on different biological pathways, and their combined application may offer an effective treatment option for patients needing further LDL-C reduction (29, 34). While PCSK9 inhibitors have shown significant efficacy in lowering LDL cholesterol (LDL-C) and reducing cardiovascular events, there are still notable research gaps, particularly regarding long-term efficacy and safety, immunogenicity, deeper mechanistic insights, and population-specific outcomes are essential areas for future research on PCSK9 inhibitors. Addressing these gaps will provide a more comprehensive assessment of PCSK9 inhibitors' clinical value and guide their optimal use in managing cardiovascular diseases.

Percutaneous coronary intervention (PCI) is a primary treatment for coronary artery disease, yet managing post-PCI patients to prevent recurrent cardiovascular events remains a clinical challenge (35). Central to this is achieving and maintaining low levels of LDL-C, which is crucial for reducing the risk of further events (33). Despite the use of statins, many patients fail to reach target LDL-C levels, indicating a need for alternative therapies. PCSK9 inhibitors have shown promise in further reducing LDL-C levels in patients post-PCI, including those who do not adequately respond to statins (30). Clinical studies suggest that these inhibitors can significantly decrease cardiovascular mortality and the risk of repeat PCI, beyond the effects of standard treatments (30). Implementing PCSK9 inhibitors in clinical practice requires a careful evaluation of each patient's LDL-C levels, cardiovascular risk profile, statin tolerance, and economic considerations (36). They are particularly recommended for high-risk patients who have not met LDL-C targets with maximal statin therapy (36, 37). As PCSK9 inhibitors become more established in post-PCI care, the need for long-term data on their safety and efficacy is paramount to ensure optimal patient outcomes.

Peripheral Artery Disease (PAD) is a common vascular condition that, despite traditional treatments such as lifestyle modifications, antiplatelet medications, and statins, many patients continue to experience uncontrolled symptoms (38, 39). PCSK9 inhibitors have emerged as a promising therapeutic option for this patient population. Evidence suggests that these inhibitors not only significantly reduce LDL-C levels but also lower the risk of cardiovascular events in PAD patients (40). Specifically, analyses have shown that PAD patients treated with PCSK9 inhibitors experience a reduced risk of such events compared to untreated counterparts (41). While preliminary, research hints at the potential for PCSK9 inhibitors to enhance walking capacity and overall quality of life in PAD patients, possibly by retarding atherosclerotic progression and improving blood flow to the lower limbs (42). However, the direct correlation between PCSK9 inhibitor therapy and these functional improvements remains understudied and requires further validation (43). Identifying the subset of PAD patients who stand to benefit the most from PCSK9 inhibitor treatment is a crucial area for future research.

Recent research has uncovered that PCSK9 inhibitors not only reduce LDL-C levels but also may positively influence endothelial function, a key early indicator of atherosclerosis and CVD (44, 45). The improvement in endothelial function could be attributed to several mechanisms: PCSK9 inhibitors' ability to lower LDL-C levels can mitigate endothelial cell damage caused by elevated LDL-C, thereby preventing endothelial dysfunction (46). Additionally, these inhibitors may downregulate the expression of inflammatory markers, reducing vascular inflammation and further supporting endothelial health (47). There is also preliminary evidence that PCSK9 inhibitors might have a direct beneficial effect on endothelial cells (48). Clinical trials have corroborated these findings, showing improved endothelial function in patients treated with PCSK9 inhibitors (49). The capacity of PCSK9 inhibitors to enhance endothelial function presents a novel therapeutic strategy in the management of CVD. Future research should focus on elucidating the precise mechanisms through which PCSK9 inhibitors exert these effects. Understanding these mechanisms is vital for optimizing the clinical application of PCSK9 inhibitors to prevent and treat CVD effectively.

In summary, PCSK9 inhibitors represent a groundbreaking advancement in lipid-lowering therapeutics, garnering significant attention and enthusiasm within the scientific community. Future research is expected to focus on several critical areas, including a comprehensive assessment of long-term safety, the development of personalized treatment regimens, the expansion of their therapeutic applications, and the exploration of novel molecular mechanisms and targets. As research progresses, PCSK9 inhibitors are anticipated to play a crucial role in the comprehensive management of dyslipidemia, aiding in both its prevention and the mitigation of its consequences. Their impact is likely to be significant in strengthening our defenses against CVD—one of the foremost global health challenges—and in enhancing the overall efficacy of therapeutic interventions.

### Limitations

Despite implementing comprehensive measures to include extensive data and ensure the study's reliability, several inherent limitations were encountered. Firstly, relying exclusively on the WoSCC database may skew results differently than if other databases were used, potentially introducing bias. Secondly, limiting the analysis to English-language studies could have omitted significant contributions available in other languages. Thirdly, variations in publication and citation patterns over time could influence the outcomes across different periods. Nevertheless, this study provides valuable insights into the subject matter and lays a groundwork for further investigation. Future studies could enhance the breadth and depth of the findings by incorporating sources from multiple databases and including research published in various languages.

## Conclusions

In this comprehensive analysis, we have delineated the temporal progression of publication and citation trends, bringing into focus the contributions of distinguished scholars such as Ray, KK from the UK. Our findings have accentuated the United States' preeminence in the arena of PCSK9 inhibitor research, as evidenced by its leading position in both the quantity of scholarly output and the magnitude of citation influence. This study has also provided an in-depth examination of the key countries, institutions, and academic journals that are pivotal to the advancement of knowledge in this field. Moreover, our meticulous analysis of keyword trends has surfaced critical research focal points, which serve as a beacon for future investigative endeavors. Researchers are encouraged to prioritize long-term safety studies, explore non-traditional pathways like inflammation, and conduct targeted trials for special populations, such as those with diabetes or renal disease, to enhance treatment outcomes.

## Data Availability

The original contributions presented in the study are included in the article/Supplementary Material, further inquiries can be directed to the corresponding author/s.
