# Supplementary material for: Exploring research trends and hotspots on PCSK9 inhibitor studies: a bibliometric and visual analysis spanning 2007 to 2023
Source: Front Cardiovasc Med. 2024 Nov 22;11:1474472. doi: 10.3389/fcvm.2024.1474472 (PMC11621103; doi:10.3389/fcvm.2024.1474472)
Supplement: Supplementary file 1 [file Table1.docx]

| Authors | Country | Np | Nc | H-index | ACN |
| --- | --- | --- | --- | --- | --- |
| Ray, KK | UK | 62 | 6,137 | 32 | 98.98 |
| Pordy, R | USA | 55 | 7,268 | 31 | 132.15 |
| Sabatine, MS | USA | 53 | 9,983 | 36 | 188.36 |
| Wasserman, SM | USA | 53 | 12,033 | 37 | 227.04 |
| Giugliano, RP | USA | 51 | 9,354 | 32 | 183.41 |
| Raal, FJ | South Africa | 48 | 7,379 | 25 | 153.73 |
| Jukema, JW | Netherlands | 41 | 5,339 | 29 | 130.22 |
| Schwartz, GG | USA | 41 | 3,553 | 22 | 86.66 |
| Somaratne, R | USA | 41 | 5,810 | 27 | 141.71 |
| Bhatt, DL | USA | 39 | 3,551 | 20 | 91.05 |

**Supplementary Table 1**. Top 10 productive authors.

Note: Np: number of publications; Nc: number of citations without self-citations; ACN: average citation number.
