# Supplementary material for: Exploring research trends and hotspots on PCSK9 inhibitor studies: a bibliometric and visual analysis spanning 2007 to 2023
Source: Front Cardiovasc Med. 2024 Nov 22;11:1474472. doi: 10.3389/fcvm.2024.1474472 (PMC11621103; doi:10.3389/fcvm.2024.1474472)
Supplement: Supplementary file 2 [file Table2.docx]

| **Country** | **Np** | **Nc** | **H-index** | **ACN** |
| --- | --- | --- | --- | --- |
| USA | 776 | 34,289 | 93 | 44.19 |
| England | 260 | 18,659 | 60 | 71.77 |
| China | 244 | 4,620 | 28 | 18.93 |
| Italy | 241 | 10,112 | 43 | 41.96 |
| France | 208 | 13,895 | 54 | 66.80 |
| Canada | 197 | 15,369 | 54 | 78.02 |
| Germany | 184 | 12,549 | 48 | 68.20 |
| Netherlands | 169 | 16,225 | 56 | 96.01 |
| Australia | 162 | 14,970 | 50 | 92.41 |
| Switzerland | 113 | 7,452 | 40 | 65.95 |
| **Subject** |  |  |  |  |
| Cardiovascular System Cardiology | 812 | 23,281 | 81 | 28.67 |
| Pharmacology Pharmacy | 483 | 6,218 | 38 | 12.87 |
| General Internal Medicine | 191 | 15,950 | 33 | 83.51 |
| Endocrinology Metabolism | 142 | 3,193 | 30 | 22.49 |
| Biochemistry Molecular Biology | 135 | 2,437 | 23 | 18.05 |
| Research Experimental Medicine | 103 | 1,344 | 20 | 13.05 |
| Health Care Sciences Services | 44 | 277 | 9 | 6.30 |
| Chemistry | 39 | 387 | 12 | 9.92 |
| Science Technology Other Topics | 36 | 689 | 14 | 19.14 |
| Nutrition Dietetics | 30 | 445 | 11 | 14.83 |

**Supplementary Table 2.** Top 10 most prolific countries and subjects.

Note: Np: number of publications; Nc: number of citations without self-citations; ACN: average citation number.

Note: Np: number of publications; Nc: number of citations without self-citations; ACN: average citation number.
