# Supplementary material for: Exploring research trends and hotspots on PCSK9 inhibitor studies: a bibliometric and visual analysis spanning 2007 to 2023
Source: Front Cardiovasc Med. 2024 Nov 22;11:1474472. doi: 10.3389/fcvm.2024.1474472 (PMC11621103; doi:10.3389/fcvm.2024.1474472)
Supplement: Supplementary file 3 [file Table3.docx]

**Supplementary Table 3.** Top 10 most prolific institutions/journals.

| **Institutions** | **Np** | **Nc** | **H-index** | **Country** | **ACN** |
| --- | --- | --- | --- | --- | --- |
| Amgen | 164 | 15,426 | 54 | USA | 94.06 |
| Harvard University | 161 | 16,089 | 53 | USA | 99.93 |
| Sanofi Aventis | 157 | 9,395 | 44 | France | 59.84 |
| Regeneron | 142 | 9,914 | 46 | USA | 69.82 |
| Brigham and Women’s Hospital | 128 | 14,979 | 51 | USA | 117.02 |
| Imperial College London | 127 | 13,761 | 48 | UK | 108.35 |
| Harvard Medical School | 119 | 12,694 | 44 | USA | 106.67 |
| Sanofi France | 110 | 8,695 | 42 | France | 79.05 |
| Institut National De La Sante Et De La Recherche Medicale Inserm | 89 | 5,546 | 32 | France | 62.31 |
| University of Amsterdam | 87 | 10,955 | 43 | Netherland | 125.92 |
| **Journals** | **Np** | **Nc** | **H-index** | **Impact Factor** | **ACN** |
| Journal of Clinical Lipidology | 87 | 1,967 | 25 | 4.4 | 22.61 |
| Atherosclerosis | 48 | 1,125 | 18 | 5.3 | 23.44 |
| Journal of The American College of Cardiology | 44 | 6,962 | 28 | 24.4 | 158.23 |
| Current Atherosclerosis Reports | 40 | 541 | 13 | 5.8 | 13.53 |
| European Heart Journal | 38 | 3,600 | 36 | 39.3 | 94.74 |
| Circulation | 33 | 3,607 | 28 | 37.8 | 109.30 |
| Current Opinion In Lipidology | 29 | 499 | 13 | 4.4 | 17.21 |
| Journal of The American Heart Association | 28 | 785 | 14 | 5.4 | 28.04 |
| Cardiovascular Drugs And Therapy | 27 | 446 | 10 | 3.4 | 16.52 |
| Journal of Clinical Medicine | 26 | 146 | 7 | 3.9 | 5.62 |

Note: Np: number of publications; Nc: number of citations without self-citations; ACN: average citation number.
