# Supplementary material for: Exploring research trends and hotspots on PCSK9 inhibitor studies: a bibliometric and visual analysis spanning 2007 to 2023
Source: Front Cardiovasc Med. 2024 Nov 22;11:1474472. doi: 10.3389/fcvm.2024.1474472 (PMC11621103; doi:10.3389/fcvm.2024.1474472)
Supplement: Supplementary file 4 [file Table4.docx]

**Supplementary Table 4.** The top 10 cited publications.

| **Title** | **Type** | **Author** | **Journal** | **Citations** | **Year** |
| --- | --- | --- | --- | --- | --- |
| Evolocumab and Clinical Outcomes in Patients with Cardiovascular Disease | Article | Sabatine, MS et,al | New England Journal of Medicine | 3,408 | 2017 |
| 2018 AHA/ACC/AACVPR/AAPA/ABC/ACPM/ADA/AGS/APhA/ASPC/NLA/PCNA Guideline on the Management of Blood Cholesterol: Executive Summary | Review | Grundy, SM et,al | Journals of the American College of Cardiology | 2,444 | 2019 |
| Alirocumab and Cardiovascular Outcomes after Acute Coronary Syndrome | Article | Schwartz, GG et,al | New England Journal of Medicine | 1,635 | 2018 |
| Efficacy and Safety of Alirocumab in Reducing Lipids and Cardiovascular Events | Article | Robinson, JG et,al | New England Journal of Medicine | 1,511 | 2015 |
| Efficacy and Safety of Evolocumab in Reducing Lipids and Cardiovascular Events | Article | Sabatine, MS et,al | New England Journal of Medicine | 1,188 | 2015 |
| Effect of Evolocumab on Progression of Coronary Disease in Statin-Treated Patients The GLAGOV Randomized Clinical Trial | Article | Nicholls, SJ et,al | Journal of the American Medical Association | 704 | 2016 |
| Inclisiran in Patients at High Cardiovascular Risk with Elevated LDL Cholesterol | Article | Ray, KK et,al | New England Journal of Medicine | 606 | 2017 |
| Low-Density Lipoprotein Cholesterol Lowering With Evolocumab and Outcomes in Patients With Peripheral Artery Disease: Insights From the FOURIER Trial (Further Cardiovascular Outcomes Research With PCSK9 Inhibition in Subjects With Elevated Risk) | Article | Bonaca, MP et,al | Circulation | 604 | 2018 |
| Two Phase 3 Trials of Inclisiran in Patients with Elevated LDL Cholesterol | Article | Ray, KK et,al | New England Journal of Medicine | 575 | 2020 |
| PCSK9 inhibition with evolocumab (AMG 145) in heterozygous familial hypercholesterolaemia (RUTHERFORD-2): a randomised, double-blind, placebo-controlled trial | Article | Raal, FJ et,al | Lancet | 551 | 2015 |
