# Supplementary material for: Exploring research trends and hotspots on PCSK9 inhibitor studies: a bibliometric and visual analysis spanning 2007 to 2023
Source: Front Cardiovasc Med. 2024 Nov 22;11:1474472. doi: 10.3389/fcvm.2024.1474472 (PMC11621103; doi:10.3389/fcvm.2024.1474472)
Supplement: Supplementary file 5 [file Table5.docx]

**Supplementary Table 5.** Top 20 keywords in the publications.

| **Rank** | **Keyword** | **Frequency** | **Rank** | **Keyword** | **Frequency** |
| --- | --- | --- | --- | --- | --- |
| 1 | efficacy | 495 | 11 | heterozygous familial hypercholesterolemia | 274 |
| 2 | safety | 440 | 12 | familial hypercholesterolemia | 270 |
| 3 | density lipoprotein cholesterol | 353 | 13 | cholesterol | 258 |
| 4 | monoclonal antibody | 324 | 14 | subtilisin/kexin type 9 | 243 |
| 5 | evolocumab | 316 | 15 | pcsk9 inhibitors | 236 |
| 6 | ldl cholesterol | 314 | 16 | alirocumab | 234 |
| 7 | cardiovascular disease | 308 | 17 | reducing lipids | 215 |
| 8 | statin therapy | 299 | 18 | pcsk9 | 205 |
| 9 | risk | 283 | 19 | therapy | 172 |
| 10 | double blind | 280 | 20 | disease | 168 |
