# Supplementary material for: Exploring research trends and hotspots on PCSK9 inhibitor studies: a bibliometric and visual analysis spanning 2007 to 2023
Source: Front Cardiovasc Med. 2024 Nov 22;11:1474472. doi: 10.3389/fcvm.2024.1474472 (PMC11621103; doi:10.3389/fcvm.2024.1474472)
Supplement: Supplementary file 6 [file Table6.docx]

**Supplementary Table 6.** The main clusters of keywords in the publications.

| **ID** | **Label (LLR)** | **Size** | **Silhouette** | **Main keyword** |
| --- | --- | --- | --- | --- |
| #0 | Pcsk9 | 94 | 0.768 | cardiovascular disease, cholesterol, atorvastatin |
| #1 | Acute Coronary Syndrome | 90 | 0.648 | disease, metaanalysis, management |
| #2 | Familial Hypercholesterolaemia | 72 | 0.688 | cardiovascular risk, inhibition, lipid lowering therapy |
| #3 | Homozygous Familial Hypercholesterolemia | 66 | 0.76 | homozygous familial hypercholesterolemia, high risk, apolipoprotein b |
| #4 | Kexin Type 9 | 63 | 0.705 | atherosclerotic cardiovascular disease, proprotein convertase subtilisin, kexin type 9 |
| #5 | Monoclonal Antibody | 61 | 0.786 | efficacy, safety, density lipoprotein cholesterol |
| #6 | Secondary Prevention | 46 | 0.786 | secondary prevention, cost effectiveness, pcsk9 inhibitor evolocumab |
| #7 | Cardiovascular Diseases | 38 | 0.773 | diabetes mellitus, cardiovascular outcome, clinical trials |
| #8 | Pcsk9 Inhibitor | 32 | 0.838 | pcsk9 inhibitor, randomized trial, heart disease |
| #9 | Statin Intolerance | 21 | 0.835 | Atherosclerosis, ldl, reduction |
