# Supplementary material for: Exploring research trends and hotspots on PCSK9 inhibitor studies: a bibliometric and visual analysis spanning 2007 to 2023
Source: Front Cardiovasc Med. 2024 Nov 22;11:1474472. doi: 10.3389/fcvm.2024.1474472 (PMC11621103; doi:10.3389/fcvm.2024.1474472)
Supplement: Supplementary file 7 [file Table7.docx]

**Supplementary Table 7.** Top 50 keywords with the strongest citation bursts.

| **Keywords** | **Strength** | **Begin** | **End** | **2007-2023** |
| --- | --- | --- | --- | --- |
| autosomal dominant hypercholesterolemia | 10.49 | 2007 | 2017 | ▃▃▃▃▃▃▃▃▃▃▃▂▂▂▂▂▂ |
| density lipoprotein receptor | 4.52 | 2007 | 2015 | ▃▃▃▃▃▃▃▃▃▂▂▂▂▂▂▂▂ |
| ldl receptor | 4.36 | 2007 | 2015 | ▃▃▃▃▃▃▃▃▃▂▂▂▂▂▂▂▂ |
| monoclonal antibody | 34.02 | 2012 | 2017 | ▂▂▂▂▂▃▃▃▃▃▃▂▂▂▂▂▂ |
| triglyceride transfer protein | 10.17 | 2012 | 2017 | ▂▂▂▂▂▃▃▃▃▃▃▂▂▂▂▂▂ |
| heterozygous familial hypercholesterolemia | 23.21 | 2013 | 2017 | ▂▂▂▂▂▂▃▃▃▃▃▂▂▂▂▂▂ |
| serine protease | 11.49 | 2013 | 2017 | ▂▂▂▂▂▂▃▃▃▃▃▂▂▂▂▂▂ |
| randomized trial | 10.06 | 2013 | 2018 | ▂▂▂▂▂▂▃▃▃▃▃▃▂▂▂▂▂ |
| density lipoprotein cholesterol | 8.96 | 2013 | 2015 | ▂▂▂▂▂▂▃▃▃▂▂▂▂▂▂▂▂ |
| double blind | 8.35 | 2013 | 2016 | ▂▂▂▂▂▂▃▃▃▃▂▂▂▂▂▂▂ |
| subtilisin/kexin type 9 | 24.7 | 2014 | 2017 | ▂▂▂▂▂▂▂▃▃▃▃▂▂▂▂▂▂ |
| randomized controlled trial | 10.05 | 2014 | 2016 | ▂▂▂▂▂▂▂▃▃▃▂▂▂▂▂▂▂ |
| amg 145 | 7.84 | 2014 | 2017 | ▂▂▂▂▂▂▂▃▃▃▃▂▂▂▂▂▂ |
| ldl c | 7.11 | 2014 | 2017 | ▂▂▂▂▂▂▂▃▃▃▃▂▂▂▂▂▂ |
| association expert panel | 5.97 | 2014 | 2017 | ▂▂▂▂▂▂▂▃▃▃▃▂▂▂▂▂▂ |
| atorvastatin | 5.74 | 2014 | 2015 | ▂▂▂▂▂▂▂▃▃▂▂▂▂▂▂▂▂ |
| b synthesis inhibitor | 5.08 | 2014 | 2016 | ▂▂▂▂▂▂▂▃▃▃▂▂▂▂▂▂▂ |
| ester transfer protein | 4.65 | 2014 | 2016 | ▂▂▂▂▂▂▂▃▃▃▂▂▂▂▂▂▂ |
| controlled trial | 4.46 | 2014 | 2018 | ▂▂▂▂▂▂▂▃▃▃▃▃▂▂▂▂▂ |
| evolocumab amg 145 | 27.41 | 2015 | 2017 | ▂▂▂▂▂▂▂▂▃▃▃▂▂▂▂▂▂ |
| placebo controlled trial | 24.4 | 2015 | 2018 | ▂▂▂▂▂▂▂▂▃▃▃▃▂▂▂▂▂ |
| cardiovascular risk patients | 11.78 | 2015 | 2017 | ▂▂▂▂▂▂▂▂▃▃▃▂▂▂▂▂▂ |
| inhibitor alirocumab | 7.93 | 2015 | 2019 | ▂▂▂▂▂▂▂▂▃▃▃▃▃▂▂▂▂ |
| statin intolerant patients | 7.51 | 2015 | 2017 | ▂▂▂▂▂▂▂▂▃▃▃▂▂▂▂▂▂ |
| low density lipoprotein | 4.46 | 2015 | 2016 | ▂▂▂▂▂▂▂▂▃▃▂▂▂▂▂▂▂ |
| pcsk9 inhibition | 4.29 | 2015 | 2017 | ▂▂▂▂▂▂▂▂▃▃▃▂▂▂▂▂▂ |
| rationale | 4.04 | 2015 | 2018 | ▂▂▂▂▂▂▂▂▃▃▃▃▂▂▂▂▂ |
| lipoprotein cholesterol levels | 3.62 | 2015 | 2017 | ▂▂▂▂▂▂▂▂▃▃▃▂▂▂▂▂▂ |
| every 2 weeks | 3.5 | 2015 | 2017 | ▂▂▂▂▂▂▂▂▃▃▃▂▂▂▂▂▂ |
| reducing lipids | 14.48 | 2016 | 2018 | ▂▂▂▂▂▂▂▂▂▃▃▃▂▂▂▂▂ |
| cardiovascular events | 5.82 | 2016 | 2018 | ▂▂▂▂▂▂▂▂▂▃▃▃▂▂▂▂▂ |
| pooled analysis | 8.66 | 2017 | 2020 | ▂▂▂▂▂▂▂▂▂▂▃▃▃▃▂▂▂ |
| clinical trial | 5.81 | 2017 | 2018 | ▂▂▂▂▂▂▂▂▂▂▃▃▂▂▂▂▂ |
| task force | 3.46 | 2017 | 2019 | ▂▂▂▂▂▂▂▂▂▂▃▃▃▂▂▂▂ |
| pcsk9 inhibitor evolocumab | 10.92 | 2018 | 2019 | ▂▂▂▂▂▂▂▂▂▂▂▃▃▂▂▂▂ |
| cost effectiveness | 5.75 | 2018 | 2019 | ▂▂▂▂▂▂▂▂▂▂▂▃▃▂▂▂▂ |
| american college | 4.51 | 2018 | 2020 | ▂▂▂▂▂▂▂▂▂▂▂▃▃▃▂▂▂ |
| type 2 diabetes mellitus | 3.88 | 2018 | 2020 | ▂▂▂▂▂▂▂▂▂▂▂▃▃▃▂▂▂ |
| insights | 4.03 | 2019 | 2021 | ▂▂▂▂▂▂▂▂▂▂▂▂▃▃▃▂▂ |
| drug therapy | 3.56 | 2019 | 2020 | ▂▂▂▂▂▂▂▂▂▂▂▂▃▃▂▂▂ |
| angptl3 | 4.78 | 2020 | 2021 | ▂▂▂▂▂▂▂▂▂▂▂▂▂▃▃▂▂ |
| clinician | 4.11 | 2020 | 2021 | ▂▂▂▂▂▂▂▂▂▂▂▂▂▃▃▂▂ |
| inflammation | 3.66 | 2020 | 2023 | ▂▂▂▂▂▂▂▂▂▂▂▂▂▃▃▃▃ |
| lipoprotein(a) | 3.49 | 2020 | 2023 | ▂▂▂▂▂▂▂▂▂▂▂▂▂▃▃▃▃ |
| bempedoic acid | 10.59 | 2021 | 2023 | ▂▂▂▂▂▂▂▂▂▂▂▂▂▂▃▃▃ |
| percutaneous coronary intervention | 4.85 | 2021 | 2023 | ▂▂▂▂▂▂▂▂▂▂▂▂▂▂▃▃▃ |
| inclisiran | 4.55 | 2021 | 2023 | ▂▂▂▂▂▂▂▂▂▂▂▂▂▂▃▃▃ |
| peripheral artery disease | 4.37 | 2021 | 2023 | ▂▂▂▂▂▂▂▂▂▂▂▂▂▂▃▃▃ |
| mortality | 3.75 | 2021 | 2023 | ▂▂▂▂▂▂▂▂▂▂▂▂▂▂▃▃▃ |
| endothelial dysfunction | 3.50 | 2021 | 2023 | ▂▂▂▂▂▂▂▂▂▂▂▂▂▂▃▃▃ |
